# Adjunctive treatment in COVID-19 and sepsis—What did we learn?

**DOI:** 10.1007/s00063-023-01089-6

**Published:** 2023-11-15

**Authors:** Evangelos J. Giamarellos-Bourboulis

**Affiliations:** Fourth Department of Internal Medicine, ATTIKON University Hospital, National and Kapodistrian University of Athens, Medical School, 1 Rimini Street, 124 62 Athens, Greece

**Keywords:** SARS-CoV-2, Tocilizumab, Biomarkers, Immunotherapy, Mortality, SARS-CoV‑2, Tocilizumab, Biomarker, Immuntherapie, Mortalität

## Abstract

The introduction of anakinra, baricitinib and tocilizumab into the treatment armamentarium of severe coronavirus disease 2019 (COVID-19) reinforced the concept of immunotherapy for bacterial sepsis. The current review investigates how the example of COVID-19 may be extrapolated to sepsis using a three-step approach. In the first step, the clinical evidence on how the immunotherapy of COVID-19 assisted viral clearance is presented. In a second step, the indications acquired from human and animal studies on the need to employ strategies with primary effective phagocytosis in sepsis are presented. In a final step, lessons learnt from COVID-19 immunotherapy are applied for sepsis. The end result is that sepsis immunotherapy should rely on the use of biomarkers which provide information on the activation of a specific prevailing mechanism in order to enable the selection of the appropriate drug.

## Abstract (de)

Die Einführung von Anakinra, Baricitinib und Tocilizumab in das Armamentarium zur Behandlung der schweren Coronavirus-Krankheit 2019 (COVID-19) hat das Konzept der Immuntherapie für die bakterielle Sepsis verstärkt. In der aktuellen Übersichtsarbeit wird untersucht, wie das Beispiel von COVID-19 anhand eines dreistufigen Ansatzes auf die Sepsis übertragen werden kann. Im ersten Schritt wird die klinische Evidenz vorgestellt, wie die Immuntherapie mit COVID-19 die Elimination des Virus unterstützt hat. In einem zweiten Schritt werden die aus Human- und Tierstudien gewonnenen Hinweise auf die Notwendigkeit des Einsatzes von Strategien mit primär wirksamer Phagozytose bei Sepsis vorgestellt. In einem letzten Schritt werden die aus der COVID-19-Immuntherapie gezogenen Lehren auf die Sepsis angewandt. Das Endergebnis ist, dass sich die Immuntherapie bei Sepsis auf die Verwendung von Biomarkern stützen sollte, die Informationen über die Aktivierung eines bestimmten vorherrschenden Mechanismus liefern, um die Auswahl des geeigneten Medikaments zu ermöglichen.

## Introduction

Sepsis is the leading killer worldwide. A worldwide survey in 2017 revealed 48.9 million incident cases per year globally of which almost 11 million died [1]. By applying the new Sepsis‑3 definitions, it became obvious that almost 80% of hospitalized patients infected by severe acute respiratory syndrome coronavirus type 2 (SARS-CoV-2) had viral sepsis [2]. Contrary to sepsis, several drugs which modulate the immune response were registered for severe coronavirus disease 2019 (COVID-19), namely anakinra, baricitinib and tocilizumab as adjunctive treatment to antivirals. The present review aims to provide insights into how lessons learnt from the use of adjunctive treatment for COVID-19 might be applied in sepsis in order to advance the field.

## The burden of adjunctive treatment

Despite the high incidence and mortality, the global sepsis problem is ignored by most global health authorities. In response to this, five European societies, the European Sepsis Alliance, the European Society of Clinical Microbiology and Infectious Diseases, the European Society for Intensive Care Medicine, the European Society of Anesthesiology and Intensive Care and the European Society of Pediatric and Neonatal Intensive Care framed a call for action to the European Center for Disease Prevention and Control [3]. It might be asked why sepsis is so profoundly ignored. A clear-cut answer is that there is no drug registered for sepsis and all drugs administered to patients aim to eliminate the infection which triggered sepsis sequelae. In recognition of the need for antisepsis drugs, several randomized controlled trials (RCT) were conducted throughout the years 1990–2003 with conflicting results. Drotrecogin-alpha was registered for severe sepsis with an APACHE II score 25 or more following the results of the PROWESS RCT; however, when this RCT was repeated the results were negative and the drug was retracted from the market [4]. The experiences from the past generate the profound question on what we expect from adjunctive immunotherapy. There are two answers to this question: adjunctive therapy potentiates the effect of co-administered antimicrobics towards better elimination of bacteria by modulation of the immune response or adjunctive therapy attenuates the exacerbated proinflammatory phenomena to minimize the deleterious effects on the organs. In order to decide which is the mechanism of benefit, the current review is following a three-step approach. At the first step, all available evidence on how the successful immunotherapies tested for severe COVID-19 impacted on viral load is presented. Then, existing evidence on how persistence of bacteria drive outcome of sepsis is presented. At the last step, teachings of precision immunotherapy for COVID-19 are transferred to the sepsis field.

## Impact of viral elimination on the outcome of severe COVID-19

In a prospective study patients infected by SARS-CoV‑2 were split into those who achieved clearance of the virus from the nasopharyngeal swab in the first 16 days from onset of symptoms and into those who had prolonged presence of the virus lasting more than 16 days. The extent of bilateral lung involvement in the chest computed tomography was broader among patients with a long persistence of the virus. The absolute counts of CD4 lymphocytes and CD8 lymphocytes were lower among patients with a long viral persistence [5]. The key to the elimination of SARS-CoV‑2 is the lymphocytes and among them the natural killer (NK) cells. A study on 168 patients early during the pandemic proved that viral shedding was prolonged when the absolute NK cell count was less than 100 cells/mm^3^; this was associated with poor survival [6]. A positive association was found between circulating interleukin (IL) 6 and the viral load [7]. This drives the question if inhibition of the IL‑6 receptor by tocilizumab may improve viral clearance through an effect on the lymphocytes. Cumulative clinical evidence is presented in Table 1 [8–12] showing that inhibition of the IL‑6 pathway is associated with better viral clearance. The suggested mechanisms are through the increase of the absolute NK cell count or through up-regulation of the human leukocyte antigen-DR (HLA-DR) on CD14 monocytes which improves antigen presentation and virus-induced lymphopenia [11, 12]. It is even suggested that circulating IL‑6 antagonizes the pharmacokinetics of the antiviral drugs [9], so that blocking of IL‑6 may improve the delivery of the standard of care (SoC) antiviral agents to tissues.

**Table 1:** Clinical evidence for the deleterious role of interleukin (IL) 6 in persistence of viral load of SARS-CoV‑2 in severe COVID-19 and the benefits from IL‑6 inhibition

Reference	Study design	*N* of patients	Intervention	Outcome
[8]	Retrospective observational	SARS-CoV‑2 infection (*n* = 30) HIV infection (*n* = 25)	Darunavir, IL‑6 interaction	Circulating danunavir lower when IL-6 > 18 pg/ml (*p* < 0.0001)
[9]	RCT	– TCZ (*n* = 295) – Placebo (*n* = 143)	Viral load measurement daily in nasopharyngeal swab and blood	AUC of viral load (as copies/μl.hour-1) in swab: 4.07 (range 2.07–7.41)/TCZ and 4.61 (range 1.68–11.04)/placebo *HR for negative viral load in serum by day 14 with* TCZ 1.17 (0.82–1.68)
[10]	Prospective, non-randomized	– Non-TCZ (*n* = 62) – TCZ (*n* = 76)	Serial viral load measurements	Initial viral load was related to viral clearance (HR 0.56; *p* = 0.01) Median time to seropositivity 14 days in TCZ vs. 17 days in non-TCZ (*p* = 0.017)
[11]	Prospective	– CD14 monocytes from 8 infected patients – Six treated patients	– Ex vivo treatment of CD14 monocytes with TCZ (*n* = 8) – IV treatment with TCZ (*n* = 6)	– Increase of HLA-DR expression on CD14 monocytes – Increase of absolute lymphocyte count
[12]	Prospective	– SARS-CoV‑2 infection (*n* = 30)	TCZ treatment (*n* = 5)	Decrease of NK and NKT-cells compared to healthy controls; increase of perforin(+) and granzyme(+) NK cells after TCZ

*IL* interleukin, *IV* intravenous, *HIV* human immunodeficiency virus, *HLA* human leukocyte antigen, *NK* natural killer cells, *RCT* randomized controlled trial, *TCZ* tocilizumab, *SARS-CoV‑2* severe acute respiratory syndrome coronavirus type 2, *HR* hazard ratio, *AUC* area under the curve

In the SAVE-MORE RCT [13, 14] which led to the approval of anakinra for patients at risk for progression into severe respiratory failure, the primary endpoint was the allocation of the two groups of treatment (SoC and placebo versus SoC and anakinra) into the different strata of the World Health Organization clinical progression scale (WHO-CPS). The comparison between the two groups showed that patients randomized to treatment with SoC and anakinra had an odds ratio (OR) of 0.36 for worse outcome than patients treated with SoC and placebo. This means that the benefit of adjunctive anakinra treatment was exerted towards both spectra of the WHO-CPS, i.e., severe disease and death or full disease resolution with viral elimination [13, 14]. There is a double interpretation of the findings; anakinra treatment prevented disease progression and the deleterious effect of the proinflammatory phenomena and, in parallel, facilitated better viral elimination.

## Bacterial elimination and sepsis outcome

Early antibiotic treatment is considered the cornerstone of sepsis management. This is suggested in all versions of the Surviving Sepsis Campaign guidelines and it is a direct expression of the need to eliminate the bacterial pathogen in order to improve the outcomes [15]; however, contrary to COVID-19 no clinical evidence is available on whether adjunctive immunotherapy in bacterial sepsis is enhancing pathogen clearance or not; however, it is self-explanatory that if the patients do not improve in the first 72 h, the administered antibiotics need to be changed and it is confirmed [16] that appropriate antibiotic treatment is a sine qua non for sepsis management. In a propensity-matched analysis, patients with bacteremia due to enterobacteria were switched after an initial intravenous course of antibiotics to oral antibiotics. The switch was carried out provided that an appropriate source control was included, orally administered antibiotics were in vitro active against the pathogen according to antimicrobial susceptibility testing and the Pitt bacteremia score was 5 or less (*n* = 739). These patients were compared to another fully matched 739 patients completely treated with intravenous antibiotics. The mortality after 30 days was similar [17]. Some treating physicians may consider this switch from intravenous to oral treatment as risky for patients with sepsis and bacteremia; however, the lack of a difference in mortality underlines that bacterial elimination using appropriate antibiotics is the most important strategy for sepsis management.

However, clinical evidence to associate a change of bacterial load with immunomodulatory treatment is not available. The current understanding of the pathophysiology of sepsis is that bacterial recognition by the immune system leads to an initial proinflammatory response which is followed by anti-inflammatory phenomena. Scarce evidence from animal studies suggests that experimental treatment which modulates either the proinflammatory or the anti-inflammatory phase and leads to improvement in survival is also accompanied by better containment of bacteria in tissues. A recent example is experimental treatment with a recombinant antibody targeting IL-36alpha in mice which developed sepsis after cecal ligation and puncture (CLP). The CLP sepsis led to an increase of IL-36alpha, but not of IL-36beta and IL-36gamma in blood and tissues. Anti-IL-36alpha after CLP prolonged survival, decreased bacterial load and improved the macrophage capacity for phagocytosis in the tissues [18]. Intratracheal instillation of *Pseudomonas aeruginosa *in mice 5 days after CLP leads to an experimental condition of immunoparalysis greatly resembling the human situation. Mice are not able to achieve potent proinflammatory responses and the death rate depends on the instilled inoculum of *P. aeruginosa *[19]. Sepsis-induced immunoparalysis of tissue macrophages exists in mice not only in the model of pneumonia following CLP but even when the first infection is pneumonia. In this setting alveolar macrophages are not able to achieve appropriate long-term phagocytosis [20]. Our group studied a model of immunoparalysis induced in rabbits after acute pyelonephritis by *Escherichia coli*. The aim was to restore immunoparalysis with recombinant human interferon-gamma (rhIFNγ). Rabbits received amikacin antibiotic treatment and co-administration with rhIFNγ which prolonged survival, decreased circulating counts of bacteria and improved the function of splenocytes for ex vivo production of tumor necrosis factor-alpha (TNF-alpha) [21].

## Transfering the success of immunotherapy for COVID-19 to bacterial sepsis

There are several lessons from the success of adjunctive immunotherapy for severe COVID-19 which must be followed in order to increase the chances that immunomodulatory treatment succeeds in sepsis. These lessons are summarized in the next sections.

### Adjunctive immunotherapy assists pathogen elimination

The paradigm of COVID-19 supports the fact that immunotherapy needs to improve the ability of the host for bacterial clearance. When immunotherapy is administered, the host may rely either on the proinflammatory phase or on the anti-inflammatory phase. The selected immunotherapy should either attenuate proinflammation or reverse anti-inflammation. Inappropriate timing of administration of a drug which further accentuates the specific phase instead of modulating it may lead to deleterious phenomena through excess propagation of the pathogen. This drives the need for tools which may diagnose if the host is in the proinflammatory phase or in the anti-inflammatory phase.

### Trials need to be appropriately powered

Not all patients are candidates for immunotherapy. Even in the case of COVID-19 following the successful trials of anakinra [13, 14], tocilizumab [22] and baricitinib [23], several subsequent trials failed to disclose similar benefits [9, 24]. Does this mean that immunotherapy does not really work? The controversy may be explained by slight differences in the study participants. In the RECOVERY open-label platform trial, patients in need of oxygen either without a ventilator, with noninvasive ventilation (NIV) or with mechanical ventilation (MV) (46%, 41% and 13% of the participants, respectively) were randomized to treatment with tocilizumab (*n* = 2022) or placebo (*n* = 2094). The mortality after 28 days was reduced from 35% in the placebo arm to 31% in the tocilizumab arm corresponding to a 15% relative efficacy [22]. A similar benefit was not found among the less severely ill patients enrolled in the CONVACTA trial [9]. In the RECOVERY open-label platform trial, patients in need of simple oxygen and non-invasive ventilation (NIV) were randomized to treatment with baricitinib (*n* = 4148) or placebo (*n* = 4008). On average 67% were in need of simple oxygen and the rest of NIV. The primary endpoint was 28-day mortality and it was met in 12% and 14% patients, respectively (*p* = 0.028) [23]. Although this difference was significant corresponding to 13% relative efficacy, it was necessary to enrol almost 8200 patients to achieve significance. The trial was repeated in smaller patient populations (*n* = 139 receiving baricitinib; *n* = 136 receiving placebo) of which the vast majority where in need of NIV. No benefit on 28-day mortality was found [24].

In a similar way, the SAVE-MORE trial which provided the registration of anakinra for COVID-19 was double-blinded randomized and powered for 594 patients [13, 14]. A recently published open-label randomized study with only 179 participants failed to meet the primary endpoint [25]. In a similar approach it needs to be underlined that the PROWESS trial which primed the registration of drotrecogin-alpha for severe sepsis was well powered. A total of 1690 patients were randomized to treatment with placebo (*n* = 840) or drotrecogin-alpha (*n*-850) and the mortality after 28 days was 30.8% and 24.7%, respectively [26].

### The proper patient population need to be selected

**Fig. 1:**
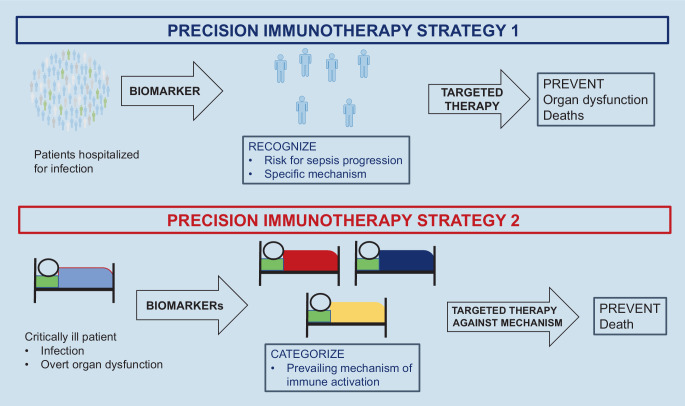
Suggested strategies of precision immunotherapy for sepsis based on lessons learnt from COVID-19. Suggested strategy 1 aims to use a biomarker in hospitalized patients with infections to predict the risk for sepsis progression and to guide precision treatment. The biomarker needs to be able to indicate the immune pathway through which the patients are at risk for deterioration. Suggested strategy 2 is applied in patients already with organ dysfunction due to sepsis. Patients need to be classified using biomarkers to different strata of immune activation. This indicates the most appropriate immunotherapeutic drug

The need for large number of study participants to demonstrate efficacy of immunotherapy is an indirect indication that not all patients will receive a benefit. The large relative benefit from anakinra treatment shown in the SAVE-MORE RCT of 64% is due to the homogeneity of the patient population in terms of the mechanism of immune activation. Study participants were hospitalized due to COVID-19 pneumonia and were in need of oxygen supplementation. All these patients had blood levels of the biomarker soluble urokinase plasminogen activator receptor (suPAR) of 6 ng/ml or more which is an indicator of the activation of IL‑1 [27]. Anakinra treatment was given at the early stages of the disease in order to prevent progression into severe respiratory failure and death. The example of the SAVE-MORE trial and of the success of tocilizumab can be extrapolated to sepsis in two strategies (Fig. 1):

- Strategy 1**: **treat patients with an infection at risk for progression to organ dysfunction through a specific mechanism which is activated. Organ dysfunction has not become apparent and a biomarker which indicates the activation of the specific inflammatory cascade to be inhibited is required. One such example is the on-going INSPIRE trial (Clinicaltrials.gov NCT05785442). This a double-blind RCT in which patients with community-acquired pneumonia (CAP) or hospital-acquired pneumonia (HAP) and circulating presepsin levels of more than 350 pg/ml are randomized to treatment with SoC and placebo or SoC and anakinra for 10 days. The primary endpoint is the progression of the patient to organ dysfunction or death. Organ dysfunction if defined as an increase of the total admission sequential organ failure assessment (SOFA) score by two or more during treatment [28].
- Strategy 2: treat patients with full-blown sepsis and overt organ dysfunction according to the prevailing pathophysiology. One such example is the on-going ImmunoSep trial (Clinicaltrials.gov NCT01990232) in six European countries (Germany, Greece, Italy, the Netherlands, Romania, Switzerland). This a double-blind, double-dummy RCT in which patients with CAP or HAP or ventilator-associated pneumonia or primary bacteremia are randomized to one arm of SoC and placebo immunotherapy or one arm of SoC and precision immunotherapy. Before randomized patients are screened for the level of immune activation by measurement of blood ferritin and of the expression of HLA-DR on CD14 monocytes. When ferritin is more than 4420 ng/ml, participants are classified into macrophage activation-like syndrome. When ferritin is 4420 ng/ml or less and the absolute count of HLA-DR receptors on CD14 monocytes is less than 5000/cell patients are classified into sepsis-induced immunoparalysis. Patients with MALS (macrophage activation-like syndrome) receive intravenous treatment three times daily with anakinra for 15 days and subcutaneous dummy placebo. Patients with sepsis-induced immunoparalysis receive subcutaneous treatment with rhIFNγ every 48 h for 15 days and intravenous dummy placebo treatment. Patients who cannot be categorized into MALS or sepsis-induced immunoparalysis cannot participate in the study [29]. The used cut-offs of ferritin and HLA-DR for patient classification have been developed by a previous phase IIa trial [30].

## Conclusion

The introduction of anakinra, baricitinib and tocilizumab for the treatment of severe COVID-10 reinforced the concept of immunotherapy in sepsis; however, it seems that it is high time that instead of testing drugs in all patients, well-characterized populations should be treated using biomarkers which identify the prevailing immune mechanism in the body of the host. This approach may maximize efficacy and bring new advances in the field.

## Declarations

This is a review article and there was no need for Ethics approval.

The supplement containing this article is not sponsored by industry.
